# Perioperative and Pathological Outcome of Nerve-Sparing Radical Cystectomy With Ileal Neobladder

**DOI:** 10.3389/fsurg.2021.652958

**Published:** 2021-03-31

**Authors:** Katharina Vogt, Christopher Netsch, Benedikt Becker, Sebastian Oye, Andreas J. Gross, Clemens M. Rosenbaum

**Affiliations:** Department of Urology, Asklepios Hospital Barmbek, Hamburg, Germany

**Keywords:** urinary bladder neoplasms, functional outcomes, bladder cancer, urinary diversion, nerve sparing

## Abstract

**Purpose:** So far, it has not been described whether the perioperative course and the pathologic outcome of patients who undergo radical cystectomy (RC) with orthotopic bladder substitution differs if nerve sparing (NS) is performed or not.

**Material and Methods:** In all, there were 472 patients who underwent RC between 2012 and 2019 at our department. We performed a retrospective analysis of 116 patients who underwent RC with ileal neobladder. We analyzed perioperative complications according to the Clavien–Dindo classification system, as well as the pathological outcome.

**Results:** Of 116 patients, 68 (58.6%) underwent RC, and 48 (41.4%) underwent NS RC. Clavien–Dindo complications ≥3b occurred in 15 (12.9%) of all patients. Only infectious complications differed among the groups [NS RC: 25 patients (52.1%) vs. RC: 20 patients (29.4%); *p* = 0.02]. There was no significant difference concerning tumor stage. Concomitant Cis was present in 24 patients (35.3%) of the RC group and in 27 patients (56.3%) of the NS RC group (*p* = 0.036). Nodal status and positive surgical margin status of the bladder tumor did not differ among the groups. In all, 42 of all male patients (45.7%) had an incidental prostatic carcinoma. Positive surgical margins concerning the prostate carcinoma occurred in six patients, with all cases in the RC group (*p* = 0.029).

**Conclusions:** Our data suggest that performing NS during RC in carefully selected patients is a safe procedure and does not impair perioperative outcome. Pathological outcome of NS RC is comparable as well.

## Introduction

Radical cystectomy (RC) is still the gold-standard intended curative therapy for muscle-invasive bladder cancer and one therapy option in recurrent high-grade non–muscle-invasive bladder cancer (1). In young and otherwise healthy patients, orthotopic bladder substitution (OBS) is often performed (2). This continent urinary diversion has several advantages. However, it is associated with significant morbidity. Moreover, OBS comes along with a significant prevalence of postoperative erectile dysfunction and urinary incontinence.

In 1982, Walsh and Donker (3) were the first to describe that impotence after radical prostatectomy (RP) occurred due to injury of the nerves of the pelvic plexus. In patients with organ-confined tumor status where RC or RP is performed, sparing the nerves of the prostate capsule was recommended in order to preserve sexual function. Over the past 30 years, there have been various approaches to improve the technique of nerve sparing (NS) for the purpose of achieving a good functional outcome. Erectile function has been ensured to be improved by NS technique (4, 5).

Besides sexual function, there is a clear benefit of NS regarding daytime and nighttime continence. Furrer et al. (4) analyzed 156 patients with NS OBS at a median follow-up of 169 months reporting that 89% of the patients were continent during daytime and 69% during nighttime. According to today's state of knowledge, NS RC should be performed whenever possible (6).

The short-term morbidity in RC is known to be high. Vetterlein et al. (7) recently revealed their high-volume analysis of RC patients where 97% of them developed more than one complication within 30 days after operation. So far, it is not described whether and how the perioperative course of patients who undergo NS RC differs from patients undergoing RC. In RP, it was demonstrated that an NS approach can be performed with comparable perioperative results (8).

Moreover, because of sparse data, there is uncertainty as to whether the pathological results of NS RC patients are better or worse (9). Based on this knowledge, the aim of our analysis was to investigate whether there are perioperative differences between NS RC and RC patients and if there is a distinction concerning the pathological outcome.

## Materials and Methods

A retrospective analysis of prospectively collected data from our department was performed, capturing 472 patients who had undergone open RC (ORC) and bilateral pelvic lymph node dissection for bladder cancer between January 1, 2012, and December 31, 2019. We excluded all patients who received an incontinent urinary diversion (*n* = 356), leaving 116 patients for final analysis. All of them received an ileal neobladder as described by Hautmann et al. (10).

NS RC in male patients was performed with a technique, modified to that initially described by Walsh and Schlegel (11). This technique includes removal of bladder, prostate, and seminal vesicles, leaving the neurovascular bundles intact. The procedure is carried out by a transperitoneal approach, using a combined ante–retrograde bladder dissection in combination with an intrafascial prostatectomy as described by Stolzenburg et al. (12).

NS RC in female patients was performed with a technique as initially described by Furrer et al. (4). The autonomic nerves of the dorsomedial bladder pedicle were spared around the cervix uteri, in the cervicovesical angle, and most of all along the ventrolateral paravaginal plane.

We analyzed clinical characteristics, time of surgery, perioperative complications during hospital stay, and pathological outcome.

Clinical characteristics were defined as age, gender, American Society of Anesthesiologists (ASA) physical status classification system, level of creatinine, and estimated glomerular filtration rate.

Perioperative complications were recorded during the hospital stay according to the Clavien–Dindo classification (CDC) system (13). We considered those complications that occurred during the hospital stay to be perioperative. We subdivided complications into gastrointestinal (mechanical and paralytical ileus, insufficient ileal anastomosis), infectious [defined as any infections that require antibiotic treatment more than the standard antibiotic regimen (e.g., pneumonia, wound infection)], cardiovascular (lung embolism, thrombosis, stroke, cardiac arrhythmia, cardiac infarction), and genitourinary complications (urinoma). Further on, we assessed the number of patients who received blood transfusions during hospital stay.

Pathological characteristics of the bladder tumor included tumor and nodal stage according to the most recent tumor, node, metastasis classification system, lymphovascular invasion, and lymph node characteristics. Pathological outcome of the prostate was assessed using the American Joint Cancer Committee 2002 staging system, and tumor grading was classified by the Gleason Grading (GS) system.

Descriptive statistics of categorical variables focused on frequencies and proportions. Means, standard deviation, medians, and ranges were reported where adequate. A χ^2^ test and *t* test were used to compare proportions and means, respectively. The distribution of GS among NS RC vs. non-NS RC patients was analyzed using the Wilcoxon rank sum test. Statistical significance was considered at *p* < 0.05. Statistical analyses were performed using SPSS version 20.0 (IBM Corp., Armonk, NY, USA).

## Results

### Patient Characteristics

Of 116 patients who were eligible for final analysis, 68 (58.6%) underwent RC, and 48 (41.4%) underwent NS RC. The rate of NS RC increased over the study period (Table 1). Of those, 22 (32.4%) female and 46 (67.6%) male patients underwent RC, whereas 2 (4.2%) female and 46 (95.8%) male patients underwent NS RC. Proportion of male patients among all NS RC patients was significantly higher (*p* < 0.001). Median age was 61 years [interquartile range (IQR), 54–68 years] and did not differ between the RC and NS RC groups (*p* = 0.818). ASA score of ≥3 was significantly more frequent in the RC group when compared to the NS RC group [26 patients (39.4%) vs. 8 patients (16.7%), *p* = 0.012]. Renal function did not differ among the groups (*p* = 0.441). Table 2 shows preoperative patient characteristics.

**Table 1:** Distribution of RC and NS RC over the study period.

**Year of RC**	**RC-NB without NS (*n*)**	**RC-NB with NS (*n*)**	**Total (*n*)**
2012	9	2	11
2013	8	4	12
2014	16	3	19
2015	13	1	14
2016	8	4	12
2017	5	5	10
2018	8	15	23
2019	1	14	15
Total	68	4 8	116

**Table 2:** Patient characteristics of 116 patients undergoing radical cystectomy with ileal neobladder.

	**All**	**RC-NB without NS**	**RC-NB with NS**	***P*-value**
Patients, *n* (%)	116 (100%)	68 (58.6%)	48 (41.4%)	—
**Sex**				
Female, *n* (%)	24 (20.7)	22 (32.4)	2 (4.2)	<0.001
Male, *n* (%)	92 (79.3)	46 (67.6)	46 (95.8)	
Age, median (IQR) (years)	61 (54–68)	62 (57–68)	59.5 (54–65)	0.818
**ASA score**				
≤2, *n* (%)	80 (70.2)	40 (60,6)	40 (83.3)	0.012
≥3, *n* (%)	34 (29.8)	26 (39.4)	8 (16.7)	
Creatinine preoperative, median (IQR) (mg/dl)	1.0 (0.8–1.0)	1.0 (0.9–1.0)	0.9 (0.8–1.0)	0.122
GFR, median (IQR) (ml/min)	84 (69–90)	81 (64–90)	88 (75–90)	0.441

### Perioperative Outcome

Median surgical time was 227 min (IQR, 195–257 min), with 239 min (201–270 min) in the RC and 212 min (182–238 min) in the NS RC group. Surgical time did not differ significantly (*p* = 0.069).

Table 3 displays the perioperative outcome. Perioperative complications occurred in 71 (61.2%) of all patients, and complications classified as Clavien–Dindo ≥3b occurred in 15 (12.9%) patients. Both overall and Clavien–Dindo ≥3b complications were comparable among the groups (*p* = 0.484 and *p* = 0.177). Gastrointestinal, cardiovascular, and genitourinary complications occurred in 15 (12.9%), 9 (7.8%), and 6 (5.2%) of all patients, respectively. There were no significant differences among the groups. Only four patients (3.4%) received blood transfusions with no significant differences between the groups (*p* = 0.722). Infectious complications occurred in 45 (38.8%) of all patients, in 20 (29.4%) patients of the RC group, and in 25 (52.1%) patients of the NS RC group (*p* = 0.02).

**Table 3:** Perioperative data of 116 patients undergoing radical cystectomy with ileal neobladder.

	**All**	**RC-NB without NS**	**RC-NB with NS**	***P*-value**
Surgical time, median (IQR) (min)	227 (195–257)	239 (201–270)	212 (182–238)	0.069
Periperative complications, *n* (%)	71 (61.2)	37 (54.4)	34 (70.8)	0.484
Clavien–Dindo ≥3b, *n* (%)	15 (12.9)	6 (8.8)	9 (18.8)	0.117
Gastrointestinal, *n* (%)^*^	15 (12.9)	8 (11.8)	7 (14.6)	0.78
Infectious, *n* (%)+	45 (38.8)	20 (29.4)	25 (52.1)	0.02
Cardiovascular, *n* (%)#	9 (7.8)	7 (10.3)	2 (4.2)	0.224
Blood transfusion, *n* (%)	4 (3.4)	2 (2.9)	2 (4.2)	0.722
Genitourinary, *n* (%)±	6 (5.2)	2 (2.9)	4 (8.3)	0.197

* *Gastrointestinal complications: mechanical and paralytical ileus and insufficient ileal anastomosis*.

+*Infectious complications: any infections that require antibiotic treatment more than the standard antibiotic regimen (e.g., pneumonia, wound infection)*.

# *Cardiovascular complications: lung embolism, thrombosis, stroke, cardiac arrythmia, and cardiac infarction*.

±*Genitourinary complications: urinoma*.

### Pathological Outcome

Pathological outcome is shown in Table 4. Most of the patients had a bladder tumor stage of ≤pT2 [83 patients (71.6%)]. There were no significant differences among the groups (*p* = 0.834). Concomitant carcinoma in situ (CIS) occurred in 24 patients (35.3%) of the RC and in 27 patients (56.3%) of the NS RC group (*p* = 0.036). Nodal status (*p* = 0.458) and positive surgical margin status of the bladder tumor (*p* = 0.949) did not differ among the groups. In all, 42 of all male patients (45.7%) had an incidental prostatic adenocarcinoma. There was no significant difference between the groups (*p* = 0.296). Distribution of GS did not differ among the groups, with GS 3+3 being the most common grade [25 patients (59.5%); *p* = 0.414]. Local stage of prostate cancer was comparable among groups (*p* = 0.834). No patient had lymphonodal invasion of prostatic cancer at time of surgery. Positive surgical margins occurred in six patients, with all cases in the RC group (*p* = 0.029).

**Table 4:** Pathologic outcome of 116 patients undergoing radical cystectomy with ileal neobladder.

	**All**	**RC-NB without NS**	**RC-NB with NS**	***P*-value**
**Pathology bladder**				
≤pT2, *n* (%)	83 (71.6)	47 (69.1)	36 (75.0)	0.489
≥pT3, *n* (%)	33 (28.4)	21 (30.9)	12 (25.0)	
Concomitant Cis, *n* (%)	51 (44)	24 (35.3)	27 (56.3)	0.036
pN+, *n* (%)	23 (20.4)	15 (22.7)	8 (17.0)	0.458
PSM, *n* (%)	5 (4.3)	3 (4.4)	2 (4.2)	0.949
**Pathology prostate**				
Prostate cancer, *n* (%)+	42 (45.7)	24 (52.2)	18 (39.1)	0.296
Gleason score 3+3, *n* (%)*	25 (59.5)	16 (66.7)	9 (50.0)	0.414
Gleason score 3+4/4+3, *n* (%)*	16 (38.1)	8 (33.3)	8 (44.4)	
Gleason score ≥4+4, *n* (%)*	1 (2.4)	0	1 (5.6)	
≤ pT2, *n* (%)*	40 (95.2)	23 (95.8)	17 (94.4)	0.834
≥ pT3, *n* (%)*	2 (4.8)	1 (4.2)	1 (5.6)	
pN+, *n* (%)*	0	0	0	—
PSM, *n* (%)*	6 (14.3)	6 (25.0)	0	0.029

+*Percentage only of male patients*.

* *Percentage of all prostate cancer patients*.

## Discussion

Even though OBS is a well-established treatment for muscle-invasive bladder cancer, it has negative impact on the patient's quality of life, especially regarding sexual function and urinary continence. Continence and erectile dysfunction after ileal OBS are influenced by several intraoperative factors. In OBS, recent studies showed a benefit in potency and continence rates after NS RC and recommended to perform NS RC if possible (14–17). Based on those references, the aim of our investigation was to analyze whether there are perioperative or pathological differences between patients undergoing RC and those undergoing NS RC. To the best of our knowledge, no data exist regarding the comparison of perioperative complications between NS and non-NS OBS patients.

Patients for an NS approach were selected carefully according to their preference, preoperative sexual function, tumor status, kidney function, and intraoperative situs. Our study did not find any difference between RC and NS RC in terms of perioperative complications or pathological outcome. Even though surgical time did not differ significantly between the groups, median operating room time in NS RC was shorter. This can be explained by a rather anatomical preparation in NS, assuming these patients are not suffering from a significant prostate cancer and tend to have a lower tumor stadium (<pT3). Likewise, cohort size might have led to this non-significant difference. Positive surgical margins regarding an incidental prostate tumor occurred only in the RC group.

One of the biggest concerns about NS RC is the potential risk of inadequate tumor resection and therefore an inferior oncological outcome. Hautmann et al. (9) demanded in 2005 to abandon “these new techniques” in order to have a better oncological outcome. However, in our investigation, we did not find any difference regarding the pathological outcome. In both groups, ≤pT2 was the most common tumor stage in the bladder. Positive surgical margins concerning the bladder tumor and lymphonodal status were statistically equal in both groups. Concomitant Cis was found significantly more often in patients of the NS RC group, which is most likely to be explained by the higher rate of early cystectomies in high-risk non–muscle-invasive bladder tumors in this group. The local recurrence rate in NS RC is described as lower than 20% in several studies with a median follow-up time from 21 to 202 months (14, 15, 18). According to careful patient selection, positive surgical margins regarding prostate cancer occurred only in the RC group.

Vetterlein et al. (7) demonstrated in their analysis of more than 500 RC patients that 99% of patients suffer from complications at 30 days postoperatively. Of those, 11% suffered from most severe (CDC grade ≥3b) complications requiring an intervention under general anesthesia. These findings support our analysis as 12.9% of the study cohort had a complication ≥3b according to CDC. The less severe complications differ from our findings in frequency of occurrence, which most likely is to be explained by different investigation tools. According to their work, the most common complications were genitourinary (24%), gastrointestinal (19%), and infectious complications (15%), whereas in our analysis infectious complications were the most common, followed by gastrointestinal complications. Deuker et al. (19) just recently published an analysis of 11,594 patients undergoing robot-assisted RC vs. ORC testing the effects of obesity and surgical approach on perioperative outcomes and total hospital charges. Among others, they found that obesity independently predicted overall complications and major complications. In 916 patients, ORC was performed, finding major complications in 35.4%. The significantly higher complication rate could be explained by the fact that different investigation tools were used. Similarly, it can be assumed that our patient cohort tends to be healthier, as not only patients with continent urinary diversion were included in the study.

We have not captured the functional results. However, for us, it is a main driver to perform NS RC as, over the past few years, different studies showed a benefit for attempted NS regarding potency and continence rates. Muto et al. (20) found a normal erectile function at a median follow-up of 8.5 years in 95.6% of the patients. To our clinical experience, the reported findings of Colombo et al. (18) seem to be more realistic. They found an overall satisfactory erectile function in 65.5% patients at 24-month follow-up. These conflicting findings may be seen as a result of small sample sizes at follow-up, a fewer number of studies, and different evaluation tools. Also, in terms of continence rates, attempted NS seems superior. Kessler et al. (14) were able to show that attempted NS technique had a great impact on daytime continence rate in a large single-center study. These finding were supported by Colombo et al. (18) and Wang et al. (15).

Initially, OBS was performed exclusively in men. Even today, the number of female patients with OBS is significantly lower (21). In our cohort, the rate of female patients is only 20%. There are many reasons for this. One explanation for the low rate of female patients is that data on the effect of NS RC on sexual function in women are sparse, and knowledge of preservation of the neurovascular bundle in female patients is young. Recent works analyzed the sexual function after conventional RC. The studies of Volkmer et al. (22) and Zippe et al. (23) showed a significant decrease in female sexual function and continence rate after RC. Bhatta Dhar et al. (21) compared the sexual function in female patients with and without attempted NS, showing that the sexual function was preserved in patients who received neurovascular preservation.

In summary, our data and the given literature suggest that performing NS during RC in carefully selected patients is a safe procedure. To our knowledge, we are the first to present perioperative data regarding NS OBS, showing that the perioperative outcome is comparable, as well as the pathological outcome.

These results are likely to count for robot-assisted RC, too. Recent studies showed no difference between open, laparoscopic and laparoscopic, robot-assisted RC (24, 25). However, further studies are needed to evaluate NS in the laparoscopic RC and laparoscopic, robot-assisted RC setting.

Even though we present a large single-center analysis, there are some limitations to our study that should be mentioned. Statistical bias may occur as this is a retrospective analysis based on non-randomized data. We found a selection bias in our groups because in the NS RC group patients were healthier than were the patients in the RC group. Additionally, up until now, there is no follow-up examination to this study. Therefore, we are unable to demonstrate the influence of NS technique on functional or oncological outcome. According to the low number of female patients in our analysis, our results need to be treated with caution when it comes to the results in women.

Our findings suggest that NS RC with OBS is a safe procedure. Perioperative and pathological outcome is comparable to the patients who received a non-NS RC. We recommend performing NS in order to improve functional outcomes after cystectomy in selected patients.

## Data Availability Statement

The raw data supporting the conclusions of this article will be made available by the authors, without undue reservation.

## Author Contributions

KV: project development, data collection, data management, and manuscript writing. CN: manuscript editing and data analysis. BB: manuscript editing and data management. SO: data collection and data management. AG: project development and manuscript editing. CR: project development, data management, data analysis, and manuscript editing. All authors contributed to the article and approved the submitted version.

## Conflict of Interest

The authors declare that the research was conducted in the absence of any commercial or financial relationships that could be construed as a potential conflict of interest.
